# The Bioactivity and Photocatalytic Properties of Titania Nanotube Coatings Produced with the Use of the Low-Potential Anodization of Ti6Al4V Alloy Surface

**DOI:** 10.3390/nano7080197

**Published:** 2017-07-26

**Authors:** Aleksandra Radtke, Adrian Topolski, Tomasz Jędrzejewski, Wiesław Kozak, Beata Sadowska, Marzena Więckowska-Szakiel, Magdalena Szubka, Ewa Talik, Lars Pleth Nielsen, Piotr Piszczek

**Affiliations:** 1 Faculty of Chemistry, Nicolaus Copernicus University in Toruń, Gagarina 7, 87-100 Toruń, Poland; topolski@umk.pl (A.T.); piszczek@chem.umk.pl (P.P.); 2 Nano-Implant Ltd. Gagarina 5, 87-100 Toruń, Poland; 3 Faculty of Biology and Environmental Protection, Nicolaus Copernicus University in Toruń, Lwowska 1, 87-100 Torun, Poland; tomaszj@umk.pl (T.J.); wkozak@umk.pl (W.K.); 4 Faculty of Biology and Environmental Protection, University of Łódź, Banacha 12/16, 90-237 Łódź, Poland; beata.sadowska@biol.uni.lodz.pl (B.S.); marzena.wieckowska@biol.uni.lodz.pl (M.W.-S.); 5 A. Chełkowski Institute of Physics, University of Silesia, Uniwersytecka 4, 40-007 Katowice, Poland; magdalena.szubka@us.edu.pl (M.S.); Ewa.Talik@us.edu.pl (E.T.); 6 Tribology Centre, Danish Technological Institute, Kongsvang Allé 29, 8000 Aarhus C, Denmark; lpn@teknologisk.dk

**Keywords:** titania nanotubes, anodic oxidation, biointegration, antibacterial properties, photocatalytic activity

## Abstract

Titania nanotube (TNT) coatings were produced using low-potential anodic oxidation of Ti6Al4V substrates in the potential range 3–20 V. They were analysed by X-ray diffraction (XRD), Raman spectroscopy, X-ray photoelectron spectroscopy (XPS), and scanning electron microscopy (SEM). The wettability was estimated by measuring the contact angle when applying water droplets. The bioactivity of the TNT coatings was established on the basis of the biointegration assay (L929 murine fibroblasts adhesion and proliferation) and antibacterial tests against *Staphylococcus aureus* (ATCC 29213). The photocatalytic efficiency of the TNT films was studied by the degradation of methylene blue under UV irradiation. Among the studied coatings, the TiO_2_ nanotubes obtained with the use of 5 V potential (TNT5) were found to be the most appropriate for medical applications. The TNT5 sample possessed antibiofilm properties without enriching it by additional antimicrobial agent. Furthermore, it was characterized by optimal biocompatibility, performing better than pure Ti6Al4V alloy. Moreover, the same sample was the most photocatalytically active and exhibited the potential for the sterilization of implants with the use of UV light and for other environmental applications.

## 1. Introduction

The considerable progress within the field of biomaterials and their medical applications is a result of intensive development of materials science. There are numerous biomaterials that can be used in the human body, including metals, alloys, ceramics, synthetic, or natural polymers [1,2,3,4,5,6,7]. However, titanium (Ti) and titanium alloys are considered to be some of the most significant biomaterials due to their resistance towards body fluid effects, high corrosion resistance, great tensile strength, flexibility and biocompatibility. So far, they are the most widely used materials in implantology [8,9,10,11,12].

The responsibility for the biocompatibility of titanium and its alloys is attributed to the formation of a chemically stable and highly adherent thin protective passivation film of titanium oxide. The natural passivation oxide layer on titanium has a thickness about 3–8 nm and is formed spontaneously in the presence of air or oxidizing media. This is also the case in biological systems, where a bioliquid surrounds the metal [13,14,15,16,17]. In the case of implants, the stoichiometric defects and low stability of this film can lead to their delamination and loosening [18,19,20]. Therefore, it becomes necessary to make the biocompatible coating permanently bond with the surface of implants. The fabrication of titania coatings of a specified stoichiometry and morphology by the electrochemical anodization of Ti/Ti alloy surfaces is a way to achieve this aim [21,22,23,24,25,26]. This simple and inexpensive method can lead to the formation of titania nanotube coatings of desired and beneficial structure, morphology, dimensions (aspect ratio; hole diameter versus length of the nanotubes), as well as optimized physicochemical properties. According to previous reports, titania nanotube coatings are produced mainly in the anodic oxidation processes using 20 V or higher potential, and usually, they are annealed in order to obtain crystalline TiO_2_ layers.

Taking into account the economic considerations and the noticeable general trend towards the use of energy-efficient and time-efficient processes, we have focused on titania nanotube coatings (TNT) in the present study, which were produced on the surface of Ti6Al4V alloy, using possibly low potentials, i.e., 3–20 V at short process time below 20 min and without subsequent annealing. On the other hand, we took into consideration the interaction between biomaterials and the microorganisms, since foreign body-associated infections (FBAIs) are still one of the most frequent and dangerous complications of modern implantology [27,28,29]. *Staphylococci* with their wide repertoire of surface adhesion and easy ability to form biofilm are among the microorganisms that most frequently may result in infections [30,31]. It was therefore apparent for the authors that modern implant systems should, if possible, not only actively participate in the integration with the bone of the recipient, but should also prevent microbial adhesion, biofilm formation and massive inflammation after the implantation.

To achieve this goal, it means to obtain the coatings, which possess the optimal ability to osseointegrate as well as antibacterial activity (without enriching them with additional agents, such as silver nanoparticles or antibiotics), the optimization of TNT fabrication processes has been carried out, and the results of the studies on above issues are the main part of this paper. Moreover, the studies on the correlation between the antimicrobial properties and the photocatalytic activity of TNT coatings have been included into the publication in order to present their potential application in UV sterilization of implants surface.

## 2. Results

**Figure 1:**
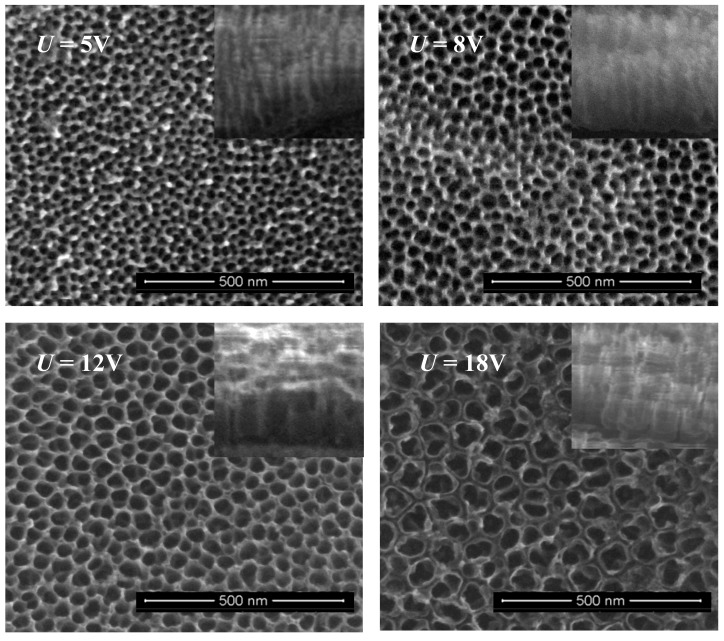
SEM (Scanning Electron Microscopy) images of the surface morphology and the cross section of titania nanotube (TNT) coatings on the surface of Ti6Al4V foil, produced at 5 V, 8 V, 12 V, and 18 V. Cross sections of the TNT coatings are illustrated as inserts.

The coatings consisting of vertically aligned titania nanotubes (TNT) were produced on the Ti6Al4V surface using the electrochemical anodization technique and known procedure [32]. Samples were produced in the potential range 3–20 V at room temperature, during a 20 min anodization process, in the presence of 0.3 wt. % aqueous hydrofluoric acid solution. Coatings obtained at the mentioned conditions were denoted as TNT3-TNT20. Analysis of TNT3-TNT20 SEM (Scanning Electron Microscopy) images revealed that uniform nanotube coatings of the same tube length (approximately 150–200 nm), open at the top and closed at the bottom, without cracks and gaps, were formed (Figure 1).

The results of BET (Brunauer–Emmett–Teller) investigations of TNT coatings produced on titanium alloy substrates showed that the value of the surface area of these coatings was decreasing as the applied anodization voltage was increasing, and were equal to 18.3, 16.8, 12.1, and 10.2 m^2^/g for layers anodized at 4 V (TNT4), 6 V (TNT6), 15 V (TNT15), and 20 V (TNT20), respectively. The above findings were in good correlation with the data obtained for TNT produced on commercially pure titanium foils [33].

### 2.1. Structural Characterization of TNT Coatings

Figure S1 in Supplementary Materials presents X-ray diffraction (XRD) patterns and Raman spectra of TNT coatings formed on the surface of titanium Ti6Al4V alloy. According to these data, materials produced on the surface of Ti6Al4V substrates, using the low potential anodic oxidation (3–20 V), were amorphous since no fingerprints of crystalline titanium dioxides could be seen neither in the XRD patterns nor in the Raman spectra.

In order to determine the nature of the oxide layer, the composition and the structure of produced TNT coatings were studied using X-ray photoelectron spectroscopy (XPS). The obtained data for as-received samples are presented in Table 1 and on Figure S2. Two peaks, which were found at binding energies of 459.0 eV and 464.8 eV, respectively, were attributed to titanium, Ti(2p_3/2_) and Ti(2p_1/2_) [34,35,36]. The splitting between the above-mentioned p-core levels is 5.8 eV (Table 1), which indicates the presence of a normal Ti^4+^ state in produced TNT coatings. The use of the deconvolution method revealed that the O(1s) peak can be composed of four (as it is visible for TNT4) or three (for TNT5-TNT18) components. The first component found at ~530.3 eV is attributed to O^2−^ in the Ti–O bond of TNT coatings. The second component located between 531.6 and 532.0 eV corresponds to oxygen of surface –OH groups. In this case, the splitting between the peaks is assigned to oxide species (TiO_2_) and hydroxyl oxygen is 1.3–1.8 eV, and it is consistent with previous reports [34]. The components, which were found in the range 532.7–533.7 eV have been assigned to oxygen of water molecules adsorbed on the TNT oxidized surface (Table 1) [37,38]. The deconvolution of the O(1s) peak of TNT4 revealed the presence of a fourth component at 533.7 eV, which was attributed to the physically adsorbed water molecules on the surface of TNT layer [39,40]. Moreover, peaks, which were found at 285.0 eV (C–H/C–C), 286.4 eV (C–O), 289.0 eV (C=O), have been assigned to adsorbed carbon oxide and organic contaminants.

### 2.2. The Wettability and the Roughness of TNT Coatings

**Figure 2:**
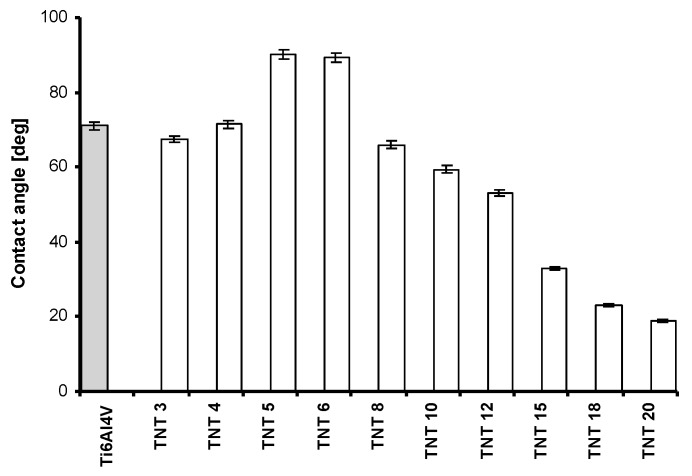
Results of wettability studies of TNT3-TNT20 samples.

**Figure 3:**
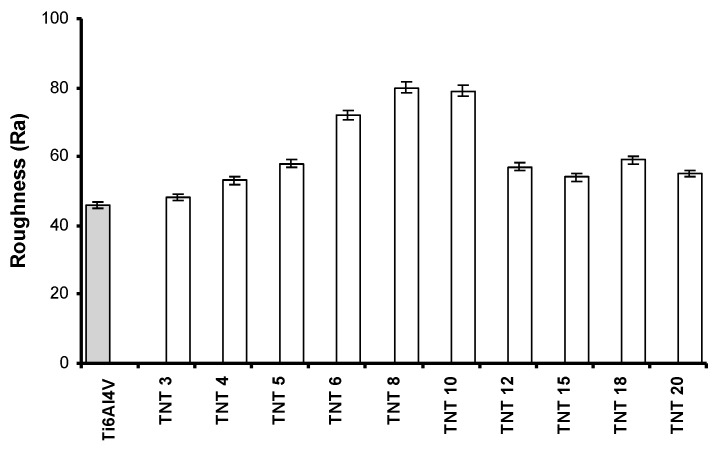
The surface roughness of TNT coatings determined by atomic force microscopy (AFM) data analysis.

The nature of hydrophobic and hydrophilic forces plays an important role both for the biological activity (impact on the cell adhesion and proliferation), as well as for the photocatalytic activity. Figure 2 shows the results of the wettability studies. They revealed the clear hydrophobic character of TNT5 and TNT6 samples. In both cases, the contact angle (θ) was close to 90 degrees and it was significantly higher in comparison to other TNT samples. The roughness of the produced coatings is another parameter influencing their biological and photocatalytic activity. Analysis of data presented in Figure 3 revealed that the roughness of TNT6-TNT10 layers is larger as compared to pure titanium alloy, and furthermore, larger than the roughness observed for TNT3-TNT5 and TNT12-TNT20 coatings.

### 2.3. Biological Activity of TNT Coatings Produced on the Surface of Ti6Al4V Foil

**Figure 4:**
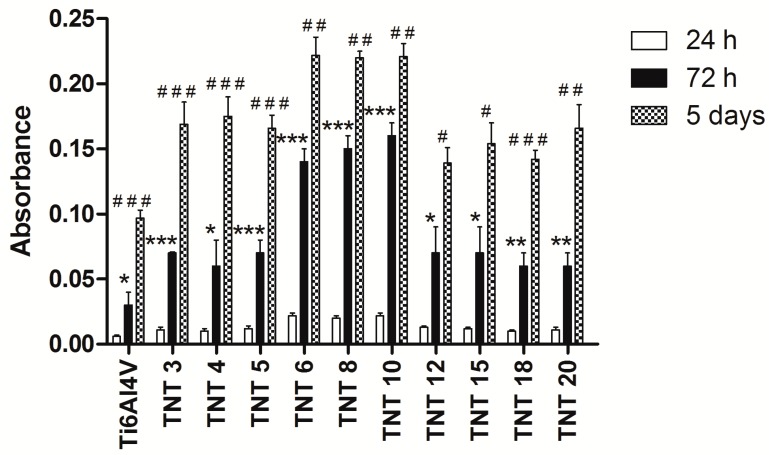
The effect of the incubation time on the murine L929 fibroblasts adhesion (after 24 h) and proliferation (after 72 h and 5 days) on the surfaces of Ti6Al4V alloy foils, modified by TNT, detected by MTT assays. The absorbance values are expressed as means ± S.E.M. of three experiments. Asterisk indicates significant differences between the cells incubated with the respective TNT for 24 h compared to 72 h of incubation time (* *P* < 0.05, ** *P* < 0.01, *** *P* < 0.001, respectively); hash mark denotes significant differences between the cells incubated with the same TNT for 72 h in comparison to 5 days’ incubation time (^#^ *P* < 0.05, ^##^ *P* < 0.01, ^###^ *P* < 0.001, respectively).

The biocompatibility of TNT coatings produced on the Ti6Al4V foil surface (Ti6Al4V-TNT system) was evaluated based on the MTT assay results, which were related to the adhesion (measured after 24 h) and proliferation (assessed after 72 h and 5 days) of L929 murine fibroblasts (Figure 4). It is worth noticing that all the studied samples showed higher level of fibroblasts proliferation than the reference samples after 72 h as well as after 5 days’ incubation time. However, this effect was most noticeable in the case of TNT6-TNT10 samples, which consisted of densely packed nanotubes of ca. 25–35 nm in diameter.

**Figure 5:**
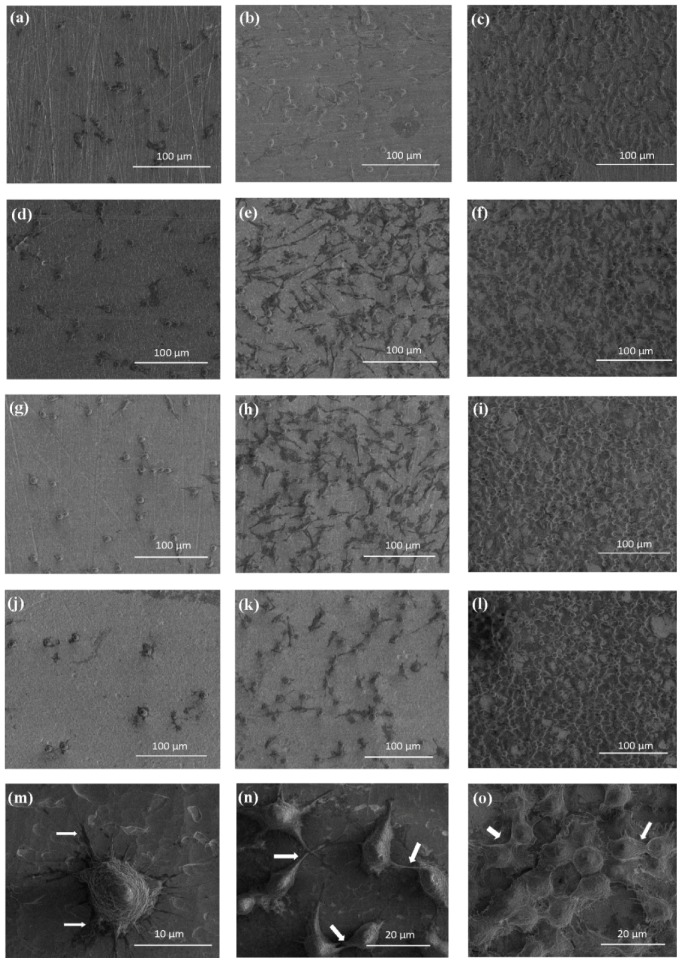
SEM images showing the cell adhesion (24 h) and proliferation (72 h and 5 days) of L929 murine fibroblasts on the Ti6Al4V alloy and TNT5, TNT10, and TNT15, after 24 h (**a**,**d**,**g**,**j**), 72 h (**b**,**e**,**h**,**k**), and 5 days (**c**,**f**,**i**,**l**) of incubation time, respectively. The arrows indicate the filopodia spread between fibroblasts incubated with TNT5 for 24 h, 72 h, and 5 days (**m**–**o**, respectively).

Figure 5 shows comparative micrographs of L929 murine fibroblasts cultured on the Ti6Al4V alloy and TiO_2_ nanotubes: TNT5, TNT10, and TNT15 for 24 h (a, d, g, j), 72-h (b, e, h, k), and 5 days (c, f, i, l) respectively. Regarding the examination by SEM, the cells cultivated on the TNT surface effectively attached to the plate surface. Importantly, the fibroblast cultured on the plates for 24 h formed filopodia, which attached the cell to the surface of the plates, but did not form them among themselves (Figure 5m). This phenomenon was observed only after 72 h and 5 days of incubation time (Figure 5n,o, respectively). Moreover, particularly after 5 days, the fibroblasts incubated on plates were crowded and were forming networks due to overgrowth of cells, which indicates that the tested plates could contribute to the proliferation of the cells. The trend shown in the SEM analysis data in Figure 5 is the same as demonstrated in MTT assay (Figure 4). Furthermore, the cells have a more rounded shape after a 24-h incubation time, whereas those fibroblasts cultured for 72 h or 5 days on the TNT became increasingly more elongated and showed a number of filopodia.

**Figure 6:**
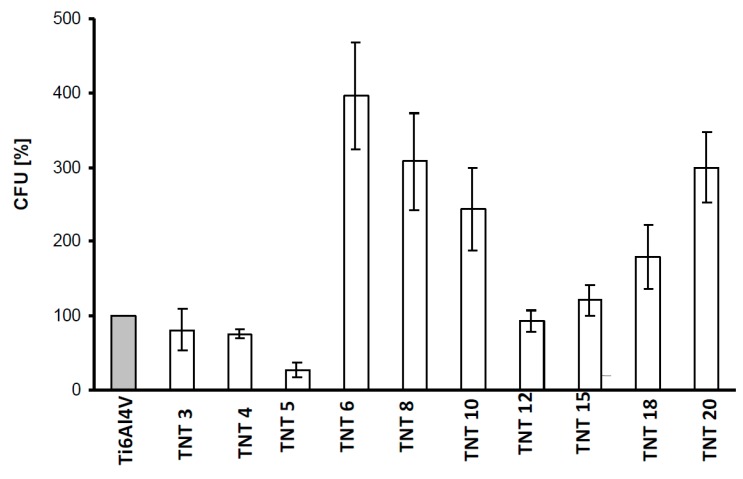
*S. aureus* ATCC 29213 aggregates/biofilm formation on the surfaces of Ti6Al4V alloy foil modified by TiO_2_ nanotubes (TNT) tested by the colony-forming units (CFU) method. The results are presented as mean percentage ± standard deviation (S.D.) of *S. aureus* CFU reclaimed after 24 h from Ti6Al4V alloy biomaterials modified by TiO_2_ nanotubes with different diameters (TNT), in comparison to bacterial CFU recovered from control (unmodified) biomaterial (Ti6Al4V) considered as 100%. Two independent sets of experiments were prepared, each in duplicate.

**Figure 7:**
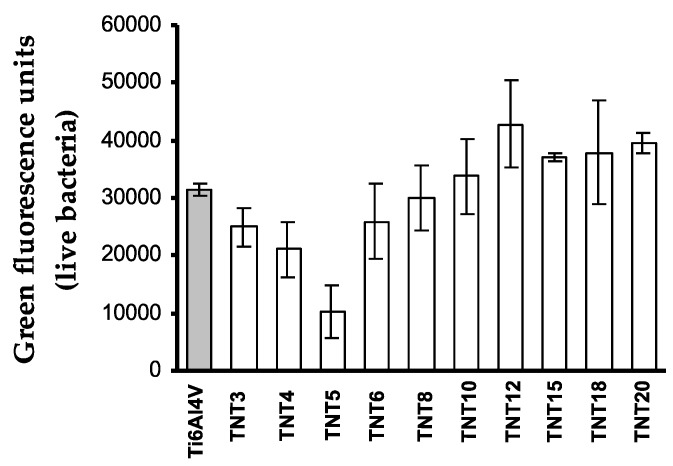
Number of live microorganisms adhered to Ti6Al4V alloy and TNT coatings, obtained in the anodic oxidation of Ti6Al4V surface.

**Figure 8:**
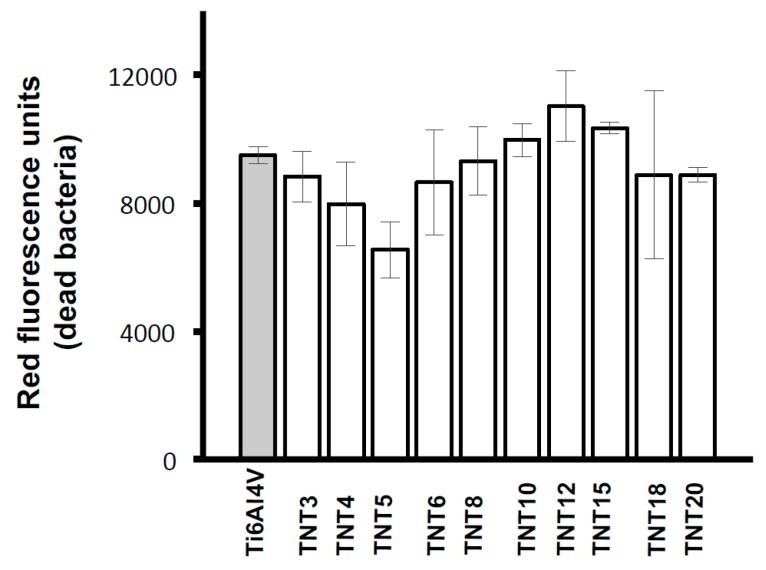
Number of dead microorganisms adhered to Ti6Al4V alloy and TNT coatings, obtained in the anodic oxidation of Ti6Al4V surface.

The antibiofilm activity of Ti6Al4V-TNT system, produced with the use of anodic oxidation in the range of potentials 3–20 V was studied against *S. aureus* ATCC 29213 using colony-forming units (CFU) and LIVE/DEAD stained assays. The contact of the bacteria with the sample surfaces lasted 24 h. Figure 6 shows that inhibitory effect for *S. aureus* ATCC 29213 biofilm formations noticed in case of TNT coatings produced at 3–5 and 12 V. LIVE/DEAD-stained BacLight Bacterial Viability Kit confirmed clear antibiofilm activity of the TNT5 sample. The lowest values of green fluorescence units (Figure 7) and red fluorescence units (Figure 8), corresponding to the number of live and dead microorganisms, respectively, obtained for the coatings produced at 5 V.

### 2.4. Photocatalytic Properties of TNT Coatings Produced on the Surface of Ti6Al4V Foil

The photodegradation of methylene blue (MB) is known in details [41]. This is why the MB photodegradation process is a good model for nanotubes photoactivity tests. The rate of MB photodegradation provides information on the photochemical activity of the studied TNT coatings produced at different anodization conditions. Additionally, it is a simple model system representative for organic water pollutant degradation. Figure S3 shows the absorbance of MB versus time dependence for the observed photodegradation process. All recorded kinetic measurements were similar in shape and could be fitted with one-exponential Equation (1),

*A_t_* = *A*_inf_ − (*A*_inf_ − *A*_0_) exp(−*k*_obs_*t*) (1)

where *A_t_*, *A*_inf_, and *A*_0_ represents absorbance in the real reaction time (*t*), infinity (inf), and start of the reaction (*t* = 0 s).

The observable rate constant is marked as *k*_obs_, and values of this parameter, designated for samples TNT3-TNT20, have been summarized in Table 2. An analysis of these data exhibits that the values of *k*_obs_ change in the narrow range from 1.63 up to 1.95 (×10^−3^ min^‒1^) and they do not seem to depend strongly on the morphology of the produced coatings and the obtained surface structure. However, the slightly higher photoactivity of samples TNT5, TNT10, TNT18 should be noted, which results in higher values of MB photodegradation rate constants (Table 2).

## 3. Discussion

**Figure 9:**
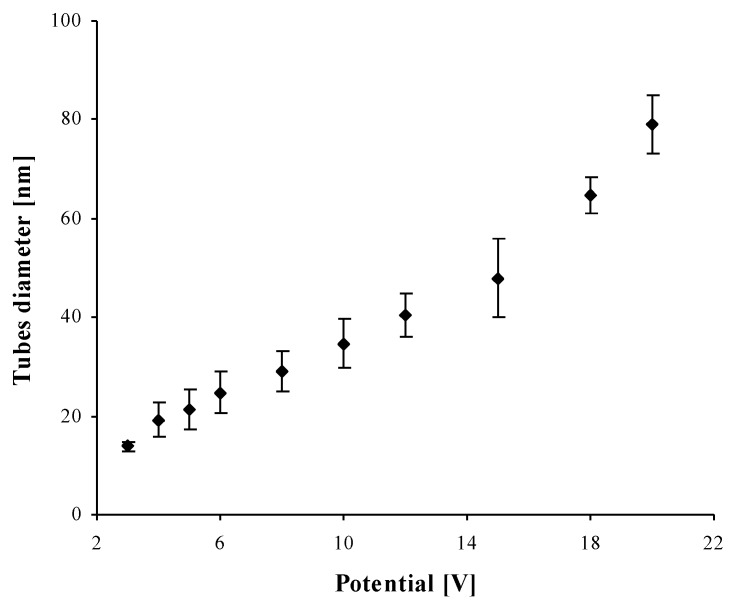
The diameter of titania nanotubes as a function of used potential.

The use of Ti6Al4V surface anodization allowed for the controlled formation of titania nanotube coatings of different tube diameters, i.e., from ca. 15 up to ca. 80 nm (Figure 9). Coatings produced between 3 V and 12 V consist of densely packed nanotubes, whereas the layers obtained at higher potentials (15–20 V) are formed by the separated nanotubes, as evident from the cross section images inserted in Figure 1. Nonlinear dependency between the used potential and the nanotubes diameter can be explained by the presence of the tubes separation processes, which proceed during the nanotubes growth process (Figure 9). An analysis of the SEM images revealed that the beginning of the nanotubes separation was observed for coatings obtained above 4 V, and the finish of the separation process was observed for samples produced at potentials higher than 12 V.

As the photo- and bioactivity of titania coatings is strongly dependent on their surface structure, it was decided to focus on the characterization of the amorphous surface structures in further details. According to McCafferty and Wightman, the metal-oxide system on the metal surface consists of three regions: (a) metal-oxide part, (b) hydroxylated part, and (c) chemisorbed water. This three-piece oxide layer is usually covered with adsorbed carbon oxides, organic contaminants from the air, as well as the physically adsorbed water [37]. The results of the XPS studies revealed that the percentage of adsorbed H_2_O molecules and OH^−^-groups on the surface of produced TNT coatings, changes depending on the condition of the anodic oxidation processes. TNT5 and TNT6, which consisted of the densely packed nanotubes of diameters of ca. 21–25 nm, are characterized by the low percentage of adsorbed H_2_O molecules and the high concentration of OH^−^-groups (Table 1). For the layer composed of nanotubes where the diameter was smaller than ca. 20 nm, (TNT4), the XPS studies confirmed the presence of chemisorbed, as well as physically adsorbed, water molecules and the low concentration of the hydroxyl groups. In turn, for nanotubes with diameters above ca. 25 nm, but still possessing the common walls, the amount of H_2_O molecules and –OH groups increases. The effect of the nanotubes’ final separation, which was noticed for TNT15, influences probably the insignificant amount of water molecules and hydroxyl groups on the surface of nanotubes.

According to earlier reports, titanium and its alloys revealed a more hydrophobic character. However, their anodic oxidation leads to the formation of more hydrophilic systems [42,43,44,45,46]. In the case of coatings composed of titania nanotubes, their hydrophilicity significantly depends on the nanotubes’ diameter, e.g., coatings composed of large diameter nanotubes are more hydrophilic. It can be explained by the fact that capillary forces of the liquid are able to facilitate water penetration into the tube interior. The TNT coatings produced at 5 V and 6 V are composed of densely packed nanotubes of diameters ca. 21–25 nm. According to the XPS data, the surface of these materials characterizes relatively low adsorption of water molecules (Table 1), which is in accord with the contact angle findings. In the comparison to TNT5 and TNT6, an increase in the hydrophilicity has been observed for both TNT surfaces having nanotubes of smaller diameters (below ca. 21 nm), as well as for TNT surfaces having larger nanotube diameters (above ca. 25 nm). The further increase in the hydrophilicity of the produced coatings is associated with the increase of the nanotube diameter, according to the trend seen by other authors in the literature [47]. The rapid increase in the TNT coatings hydrophilicity for materials produced between 12 and 20 V is associated with the separation process of nanotubes, which proceeds on the substrate surface (Figure 1 and Figure 9). Considering the obtained results, it should be noted that the nanotube diameters and their separation are the main factors influencing the wettability properties of the studied TNT coatings.

We have previously shown that the adhesion and the proliferation of fibroblasts on the surface of Ti-TNT system were significantly higher than on the surface of pure non-oxidized titanium [32]. According to literature reports, the use of Ti6Al4V alloy as a substrate offers much better physical and mechanical properties than pure titanium, as well as excellent biocompatibility [48]. The results of the studies of Ti6Al4V-TNT system, produced by the use of various potentials (3–20 V), revealed that the adhesion and the proliferation of L929 cells were greater as compared to the nonoxidized reference sample (pure Ti6Al4V) (Figure 4). The smaller differences in the cell proliferation after 5 days (*P* < 0.01 for 72 h versus *P* < 0.05 for 5 days) may be due to the fact that the fibroblasts were crowded and formed network due to overgrowth of cells. Furthermore, the cells overgrowing the entire surface of the plates did not have enough free space for further subdivisions. This assumption was confirmed by the results of the SEM analysis (Figure 5).

In general, the results of the MTT assay confirmed the promising biocompatible properties of all produced TNT coatings. They give hope for the use of studied coatings as biomaterials in implantology, since favorable cellular interaction with their surface is crucial to the long-term success of implants [49]. Analysis of data presented in Figure 4 revealed the lack of significant differences in terms of the adhesion and the proliferation of cells on the surface of TNT3-TNT5 and also TNT12-TNT20 samples. The biointegration was most noticeable in the TNT6-TNT10 samples. The mentioned greater fibroblast cells adhesion and proliferation may be associated with the high roughness values of TNT6-TNT10 (Figure 3 and Figure 4).

The inhibitory effect for *S. aureus* ATCC 29213 biofilm formations was noticed in the TNT coatings produced at 3–5 and 12 V. It was intensified with the increase of the surface hydrophobicity and it was the strongest for TNT5. This effect was observed to be weaker for TNT12-TNT20 samples, what could be associated with the increase of the hydrophilicity for these coatings consequentially with the increase of the nanotubular diameter and their separation (Figure 2 and Figure 9). The stimulation effect of the biofilm formation, which was noted for TNT6-TNT10 samples is incomprehensible and requires further explanation. The enhanced adhesion of bacteria to the nanotubular and nanotextured surfaces, is speculated by Puckett as being a result of their amorphousness and the greater nanometer surface roughness [50]. Our studies have shown that all the produced TNT coatings were amorphous, which together with the high surface roughness of TNT6-TNT10 may lead to a significant increase in their vulnerability on bacterial attachment in comparison to the conventional Ti6Al4V non-anodized surfaces. Puckett et al. also pointed out the ambiguous role of fluoride ions, which are present on the nanotubular titanium surfaces [50]. According to them, the fluorine present on the TNT coating surface may increase the adhesion of bacteria. On the other hand, the earlier studies confirmed the antibacterial effect caused by the presence of fluorine [51,52,53]. Results of our XPS studies confirmed that TNT coatings formed during the anodization processes contain fluorine ions, and furthermore, that the fluorine content is different for TNT samples obtained at different potentials (Table 3). An analysis of the XPS data indicated that the highest fluorine concentration is observed on the TNT5 surface, which might be linked with the good antimicrobial properties observed for this coating.

The LIVE/DEAD assay also confirmed clear antibiofilm activity of TNT5, as the lowest number of live and dead bacteria (Figure 7 and Figure 8) was noticed for this coating. This indicates that TNT5 is the most active surface preventing bacterial adhesion and biofilm formation. Moreover, based on the suggestions of Puckett et al., and Palma et al. it could be suspected that the growing number of dead bacteria on the selected titanium surfaces (TNT6-TNT15), which can release an intercellular protein upon death, might become the nutrient for others and enhance further adhesion to other microorganisms [50,54]. In accordance with this, the number of live staphylococci on those surfaces is also observed to increase.

Making the discussion about the photocatalytic activity of obtained nanotube coatings we can state that, generally, the values of *k*_obs_ for TNT coatings produced on the surface of Ti6Al4V are lower in comparison to previously noticed data for TNT on Ti substrates [33]. The observed difference can be explained by the amorphousness of the present TNT as well as the presence of smaller diameters of nanotubes on alloy surfaces, in comparison to TNT layers formed on titanium substrates. Nonetheless, although TNT formed on the titanium alloy reacts slower, the difference which is not distinctly significant (the same order of magnitude) and popularity of this alloy in many applications (e.g., medical) makes TNT formed on Ti6Al4V a very interesting material, which can compete with TNT formed on the pure titanium foil, if photochemical properties are taken into account.

Among the studied Ti6Al4V-TNT systems, obtained with the use of low-potential anodic oxidation of titanium alloy, only the TNT5 surface appears to maintain an appropriate balance between the tissue biocompatibility allowing for the colonization of the host eukaryotic cells and the ability to prevent the bacterial adhesion and biofilm formation. Therefore, we can conclude that TNT5 coating is superior for biomedical applications as implants surface coating. Moreover, TNT5 is the most photoactive among the studied materials, in the degradation of MB solution, so it can be used simultaneously as active coating in the process of implant surface sterilization induced by UV light.

Biological properties of TNT6, TNT8, and TNT10, which are characterized by the highest roughness compared to other produced TNT coatings, are noteworthy. These materials revealed the best properties for the adhesion and proliferation of fibroblasts. However, at the same time they are also characterized by a surprisingly high adhesion of microorganisms and tendency to biofilm formation, which excludes their use as biomedical coatings.

It should be pointed out that the obtained results have a limitation—they are adequate for Ti6Al4V alloy, which is commonly used in maxillofacial and dental implantology. In the case of the other titanium alloy use (for example Ti6Al7Nb and Ti13Nb13Zr used in orthopaedics), the optimization process of TNT production is necessary.

## 4. Materials and Methods

### 4.1. Synthesis of TiO_2_ Nanotubes Coatings (TNT)

TiO_2_ nanotubes (TNT) were produced on the surface of Ti6Al4V alloy samples (5 mm × 70 mm, Grade 5, BIBUS METALS) using the anodic oxidation method. Before the process of anodization, the substrate samples were ultrasonically cleaned sequentially in acetone (15 min), 80% ethanol (5 min), and deionised water (15 min). The substrates were dried in an Argon stream at room temperature. The surface of the substrates were chemically etched in a 1:4:5 mixture of HF:HNO_3_:H_2_O for 30 s, cleaned with deionised water, and dried in an argon (Ar) stream. The anodization was carried out at room temperature using prepared substrate as anode, platinum wire as cathode, and 0.3 wt. % aqueous HF solution as electrolyte, according to earlier reports [32]. The applied potential was varied from 3 V up to 20 V and the anodization time *t* = 20 min. In order to purify the produced coatings, they were washed with distilled water with the addition of Al_2_O_3_ powder (averaged particle size = 50 nm) in an ultrasonic bath for 1 min, and then dried in Ar stream. Samples obtained at mentioned conditions were denoted as: TNT3-TNT20.

### 4.2. Morphological and Structural Characterization of TNT Coatings

The morphology of the produced coatings was studied using Quanta field-emission gun Scanning Electron Microscope (SEM; Quanta 3D FEG; Carl Zeiss, Göttingen, Germany; 30.0 kV accelerating voltage was chosen for SEM analysis and the micrographs were recorded under high vacuum using secondary electron detector (SE)). The surface roughness of the produced coatings was established based on atomic force microscopy studies (AFM; NanoScope MultiMode SPM System, Bruker, Billerica, MA, USA, with scanning probe Veeco Digital Instrument, measurement in the tapping mode (noncontact mode), scan area: 5 × 5 μm). The structure of the produced TiO_2_ nanotube layers (TNT) was analyzed using X-ray diffraction (PANalytical X’Pert Pro MPD X-ray diffractometer, PANalytical B.V., Almelo, The Netherlands, using Cu-Kα radiation; the incidence angle was equal to 1 deg) and Raman spectroscopy (RamanMicro 200, Perkin Elmer, Waltham, MA, USA, Exposure time 2 s; Number of exposure 20; Spectral range 200–3200 cm^−1^; number of scanned points on the sample surface −10). XPS spectra of investigated samples were obtained with monochromatized Al K_α_-radiation (1486.6 eV) at room temperature using a PHI 5700/660 ESCA spectrometer (Physical Electronics, Lake Drive East Chanhassen, MN, USA). Studies on BET-specific surface area were done using the Accelerated Surface Area and Porosimetry System ASAP 2010 (Micromeritics, Norcross, GA, USA). The samples were heated (desorbed) before measurement at 70 °C to achieve a final pressure of 0.001 mbar, over 8 h. After the desorption process, the samples were weighed and placed in a measuring station in the temperature of liquid nitrogen, in which the nitrogen adsorption isotherms were determined).

### 4.3. Wettability Measurements

The wettability of TNT coatings was investigated using a Drop Shape Analyzer—DSA100S (Krüss, Hamburg, Germany) for the contact angle measurement. Ten μL of distilled water were slowly deposited on the surface of analyzed TNT coatings using a calibrated screw-syringe. The images were recorded and the contact angles were estimated by numerically fitting of the droplet images. The value of the contact angle for each biomaterial is the average value of five measurements.

### 4.4. Cell Adhesion and Proliferation Assay on TNT Coatings

Murine fibroblasts cell line L929 (American Type Culture Collection) culture conditions were the same as described previously [32]. The effect of TNT on the cells adhesion (after 24 h) and proliferation (after 72 h and 5 days, respectively) were studied by the MTT (3-(4,5-dimethylthiazole-2-yl)-2,5-diphenyl tetrazolium bromide; Sigma Aldrich, Darmstadt, Germany) assay using the same method as it was reported in [32]. The morphology changes of L929 cells grown on the surface of TiO_2_ nanotubes coatings were analyzed using Scanning Electron Microscopy (SEM, Quanta 3D FEG; Carl Zeiss, Göttingen, Germany).

### 4.5. Microbial Aggregates/Biofilm Formation on TNT Coatings

TNT coatings on the surface of Ti6Al4V alloy substrates, prepared in the accordance with the procedure previously used [32], have been exposed to *Staphylococcus aureus* ATCC 29213 reference strain. The samples (size ~5 mm × 5 mm) of studied TNT layers and unmodified Ti6Al4V alloy (control sample) were placed into *S. aureus* suspension (OD = 0.9) for 24 h and incubated in stable conditions at 37 °C to check the formation of microbial aggregates/biofilm on the surface of TNT coatings [32]. To evaluate aggregates/biofilm formation, a CFU method was used after mechanical recovery of microbial cells from the tested surfaces, as well as LIVE/DEAD-stained BacLight Bacterial Viability kit (L/D; Invitrogen, Thermo Fisher Scientific, Eugene, OA, USA).

### 4.6. Photocatalytic Degradation of Methylene Blue (MB)

Studies on the photocatalytic degradation of methylene blue (MB) were performed using MB aqueous solution of initial concentration *c*_0_ = 1.0 × 10^−5^ M, according to the procedure written in earlier reports [33]. The kinetic calculations are based on the methodology of chemical kinetics assuming a Langmuir–Hinshelwood reaction mechanism. Taking into account a low concentration of MB, it can be assumed that a photodegradation process occurs according to the pseudo-first-order kinetics, and the kinetic equation describing changes in the MB concentration during its degradation can be expressed as below (Equation (2)):

*c_t_* = *c*_0_ exp(−*k*_obs_*t*), (2)

where *c_t_* is MB concentration after time *t*, *c*_0_ is its starting concentration, and *k*_obs_ is the observable rate constant. In the calculations, blind tests (degradation of MB with no UV and no titania samples) were taken into account.

## Figures and Tables

**Table 1:** X-ray photoelectron spectroscopy (XPS) data of selected TNT samples. The binding energies are in eV.

Sample	Ti^4+^		O^2−^	OH^−^	H_2_O	H_2_O
	Ti(2p_3/2_)	Ti(2p_1/2_)	O(1s)	O(1s)	O(1s)	O(1s)
**TNT4**	459.0	464.8	530.3 (53%)	531.6 (19%)	532.7 (17%)	533.7 (11%)
**TNT5**	459.2	465.0	530.4 (67%)	531.8 (23%)	533.1 (10%)	
**TNT6**	459.0	464.8	530.3 (66%)	531.7 (24%)	533.0 (10%)	
**TNT8**	458.7	464.7	530.4 (64%)	531.9 (25%)	533.2 (11%)	
**TNT10**	458.8	464.6	530.2 (53%)	532.0 (32%)	533.4 (15%)	
**TNT15**	459.0	464.8	530.4 (69%)	531.8 (21%)	532.8 (10%)	
**TNT18**	458.7	464.5	530.3 (66%)	532.0 (23%)	533.4 (11%)	

**Table 2:** The *k*_obs_ rate constants for methylene blue (MB) photodegradation on the TNT surfaces formed at different potentials. The results take into account blind tests (no UV and no titania samples).

	Sample	TNT3	TNT4	TNT5	TNT6	TNT8	TNT10	TNT12	TNT15	TNT18	TNT20
Rate Constant											
10^3^ × *k*_obs_ (min^−1^)		1.70 ± 0.14	1.63 ± 0.15	1.91 ± 0.13	1.62 ± 0.14	1.77 ± 0.16	1.89 ± 0.20	1.70 ± 0.16	1.80 ± 0.14	1.90 ± 0.16	1.69 ± 0.15

**Table 3:** Fluorine ions’ presence on the surface of TNT coatings based of XPS studies.

Sample	TNT4	TNT5	TNT6	TNT8	TNT10	TNT15	TNT18
F %	4.2	6.3	4.5	1.5	2.9	2.4	1.3

## Supplementary Materials

The following are available online at http://www.mdpi.com/2079-4991/7/8/197/s1, Figure S1: Results of XRD studies of TNT coatings produced at 5, 8, 12, and 18V, respectively (a) and Raman spectra of these materials (b); Figure S2: XPS spectra of selected TNT samples, O1s peaks after deconvolution process.; Figure S3: Changes of MB absorbance as a function of time with UV light illumination in the presence of TNT3-TNT20.
