# Supplementary material for: The Bioactivity and Photocatalytic Properties of Titania Nanotube Coatings Produced with the Use of the Low-Potential Anodization of Ti6Al4V Alloy Surface
Source: Nanomaterials (Basel). 2017 Jul 26;7(8):197. doi: 10.3390/nano7080197 (PMC5575679; doi:10.3390/nano7080197)
Supplement: Supplementary file 1 [file nanomaterials-07-00197-s001.pdf]

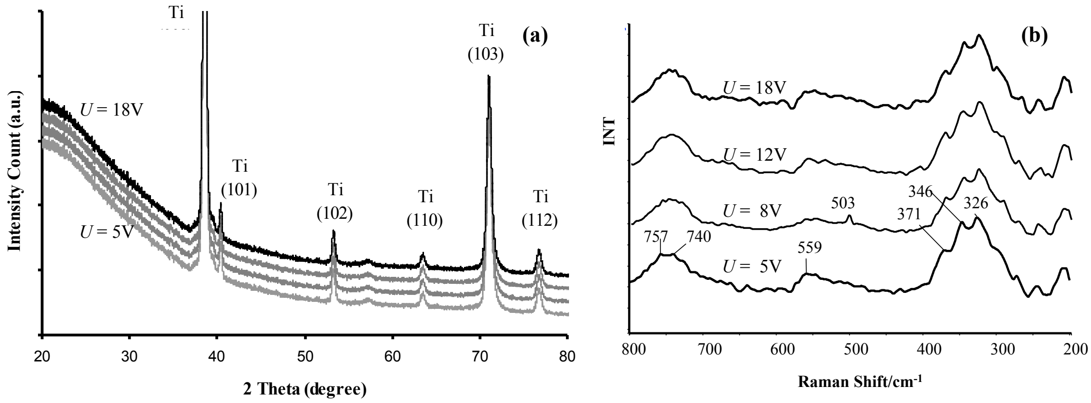

Figure S1. Results of XRD studies of TNT coatings produced at 5, 8, 12, and 18V, respectively (a) and Raman spectra of these materials (b).

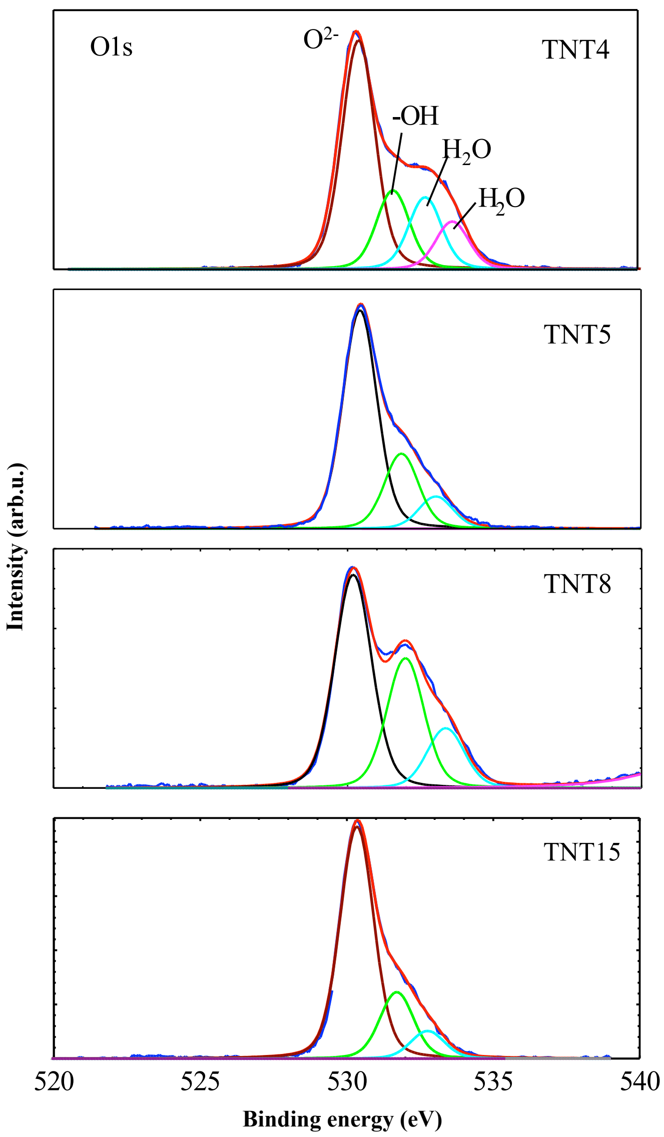

Figure S2. XPS spectra of selected TNT samples, O1s peaks after deconvolution process.

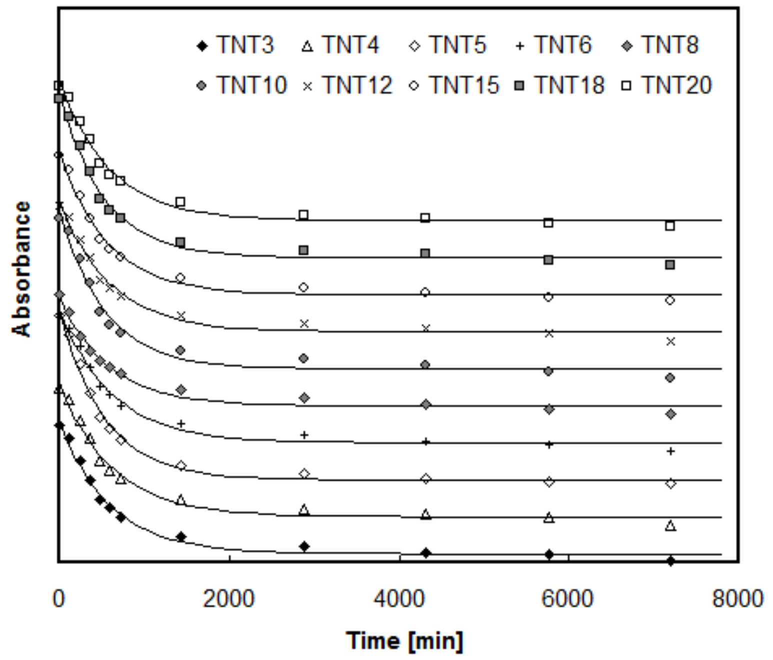

Figure S3. Changes of MB absorbance as a function of time with UV light illumination in the presence of TNT3-TNT20.
